# Administration of Extensive Hydrolysates From Caseins and *Lactobacillus rhamnosus* GG Probiotic Does Not Prevent Cow’s Milk Proteins Allergy in a Mouse Model

**DOI:** 10.3389/fimmu.2020.01700

**Published:** 2020-09-11

**Authors:** Karine Adel-Patient, Marine Guinot, Blanche Guillon, Hervé Bernard, Amina Chikhi, Stéphane Hazebrouck, Christophe Junot

**Affiliations:** ^1^Service de Pharmacologie et d’Immunoanalyse, Département Médicaments et Technologies pour la Santé (DMTS), CEA, INRAE, Université Paris-Saclay, Gif-sur-Yvette, France; ^2^Laboratoire de Physiologie de la Nutrition et de Sécurité Alimentaire, Université d’Oran 1 Ahmed Ben Bella, Oran, Algeria

**Keywords:** food allergy, prevention, hydrolyzed formulas, probiotic, cow’s milk, mouse model

## Abstract

**Background:**

Early nutrition may influence the development of food allergies later in life. In the absence of breastfeeding, hydrolysates from cow’s milk proteins (CMP) were indicated as a prevention strategy in at risk infants, but their proof of effectiveness in clinical and pre-clinical studies is still insufficient. Thanks to a validated mouse model, we then assessed specific and nonspecific preventive effects of administration of extensive hydrolysates from caseins (eHC) on the development of food allergy to CMP. The additional nonspecific effect of the probiotic *Lactobacillus GG* (LGG), commonly used in infant formula, was also assessed.

**Methods:**

Groups of young BALB/cByJ female mice were pretreated by repeated gavage either with PBS (control mice), or with PBS solution containing non-hydrolyzed milk protein isolate (MPI), eHC or eHC+LGG (eq. of 10 mg of protein/gavage). All mice were then experimentally sensitized to CMP by gavage with whole CM mixed with the Th2 mucosal adjuvant *Cholera toxin*. All mice were further chronically exposed to cow’s milk. A group of mice was kept naïve. Sensitization to both caseins and to the non-related whey protein β-lactoglobulin (BLG) was evaluated by measuring specific antibodies in plasma and specific *ex vivo* Th2/Th1/Th17 cytokine secretion. Elicitation of the allergic reaction was assessed by measuring mMCP1 in plasma obtained after oral food challenge (OFC) with CMP. Th/Treg cell frequencies in gut-associated lymphoid tissue and spleen were analyzed by flow cytometry at the end of the protocol. Robust statistical procedure combining non-supervised and supervised multivariate analyses and univariate analyses, was conducted to reveal any effect of the pretreatments.

**Results:**

PBS pretreated mice were efficiently sensitized and demonstrated elicitation of allergic reaction after OFC, whereas mice pretreated with MPI were durably protected from allergy to CMP. eHC+/-LGG pretreatments had no protective effect on sensitization to casein (specific) or BLG (non-specific), nor on CMP-induced allergic reactions. Surprisingly, eHC+LGG mice demonstrated significantly enhanced humoral and cellular immune responses after sensitization with CMP. Only some subtle changes were evidenced by flow cytometry.

**Conclusion:**

Neither specific nor nonspecific preventive effects of administration of casein-derived peptides on the development of CMP food allergy were evidenced in our experimental setup. Further studies should be conducted to delineate the mechanisms involved in the immunostimulatory potential of LGG and to clarify its significance in clinical use.

## Introduction

Type of feeding in early life may determine the propensity to develop a food allergy later in life. One of the main food allergies in infancy is a cow’s milk proteins (CMP) allergy, which affects 0.5 to 3% of children in the first year of life (1). It may be severe, persistent and have lifelong implications for health (1, 2). In most allergic children, CMP allergies can be managed using formula based on extensive hydrolysates from whey (eHW) or from caseins (eHC). Those hydrolysates contain CMP-derived small peptides with no more IgE-binding epitopes, thus preventing any elicitation of an allergic reaction in allergic infants. In clinical use, eHC formula allowed for a higher rate of tolerance acquisition to CMP compared to soya or amino acids formula (3). This effect may result from the fact that eHC still contains a large proportion of small peptides derived from caseins that may act as tolerogenic specific T-cell epitopes, or that may display non-specific immunoregulatory properties. Actually, some peptides derived from caseins possess different biological effects, such as anti-inflammatory properties (4), healing of intestinal damages, at least *in vitro* (5), and anti-microbial and immunoregulatory effects [review in (6) and (7)]. Moreover, supplementation of eHC with the probiotic *Lactobacillus rhamnosus* GG (LGG) significantly improved the observed tolerance in clinic (3, 8) and limited other allergic manifestations for up to 3 years when compared to eHC alone (9). The non-specific additional effect of LGG may result from various mechanisms, either direct (e.g., immunoregulation) or indirect (e.g., modification of microbiota composition and function, both important for intestinal barrier integrity) (10).

On the other side, the use of infant formula based on CMP hydrolysates as a diet for allergy primary prevention is a matter of high interest and debate. In the absence of breastfeeding, the use of partial or extensive hydrolysates of CMP was indicated in at-risk infants to prevent allergic sensitization to CMP and to limit the start of the “atopic march.” In this selected population, administration in the first 4 months of life of eHC or of partial hydrolysates from whey (pHW) decreased eczema incidence in the first 10 years of life when compared to standard CM formula or eHW. However, no effect on asthma or rhinitis, nor on sensitization to foods or aeroallergens, was observed (11, 12). Other interventional studies (13) or meta-analysis (14) did not support beneficial effects of CMP hydrolysates in at risk infants. A recent population-based study even demonstrated that the use of pHF at 2 months was related to higher risk of food allergy at 2 years of age, both in at risk and non-at risk infants (15). Further research on the impact of early nutrition practices using such formula for food allergy prevention is thus still of major importance in order to provide relevant and scientifically based preventive policies.

Animal models can enable the studying of the impact of postnatal nutrition on the immune responses. Two Th2-biased strains of female mice, namely C3H/HeOuJ [e.g., (16–19)] and BALB/c [e.g., (17, 20–24)], are mainly used to more specifically study food allergy and (early) oral tolerance induction, and their underlying mechanisms. In this context, by using the female BALB/c mouse model, we previously demonstrated that oral administration of the whey protein β-lactoglobulin (BLG) led to a specific tolerance that relies on the induction of regulatory T cells (Treg), and which prevents any further sensitization to this purified cow’s milk allergen (23, 25). Large peptides generated from BLG were still efficient to induce tolerance to BLG, whereas products derived from extensive hydrolysis with trypsin, leading to small peptides probably lacking T cell epitopes, were no more tolerogenic. Using an experimental model of allergy to whole CMP, we further evidenced a lower tolerogenic potential of partial hydrolysates from caseins compared to a non-hydrolyzed CMP formula (26). The tolerogenic effect was restricted to the protein source used to produce the hydrolysates, which suggests an antigenic specificity of the induced tolerance. Conversely, others have demonstrated that eHC allowed a partial prevention of allergy in a mouse model of sensitization to BLG (27), which may then rely on non-specific immunomodulatory potency of caseins-derived peptides.

In the present study, we then aimed to assess the effect of administration of eHC on a further experimental sensitization to CMP, which has never been reported. We evaluated the effect of eHC administration on sensitization to both caseins and whey proteins (BLG) in order to delineate specific from non-specific effects of caseins-derived peptides, respectively, with the nonspecific effect being the mechanism of action suggested by the outcome of clinical CMP allergy studies. We also assessed the additional non-specific effect of the probiotic LGG, a probiotic largely used in infant’s formulas.

## Materials and Methods

### Tested Materials

Non-hydrolyzed CMP (Milk protein isolate, MPI; 88% protein, containing both caseins and whey proteins), extensive hydrolysate from caseins (eHC, 85% of equivalent protein); and LGG were provided by Mead Johnson Nutrition (Evansville, IN, United States). eHC corresponds to the one found in Nutramigen formula; eHC peptide length distribution, full MS-based peptidomics description and batch-to-batch variation analysis are described in (28). Commercial whole CM (UHT, Auchan^TM^, France; 33 mg/ml of proteins) was used for experimental sensitization. For oral food challenge (OFC), commercial ultra-filtrated raw CM (Marguerite^TM^, Candia, Lyon, France) was defatted (20 min, 400 *g*, +4°C) and freeze dried to increase protein concentration. Dry powder was solubilized in water and CMP concentration adjusted at 80 mg/ml (OFC solution; BCA kit, Pierce, Thermo Scientific, Waltham, United States).

### Protocol of Tolerance Induction and CMP Sensitization in Mice

#### Ethical Considerations

All animal experiments were performed according to the European Community rules of animal care, and with specific Ethical approval from French Minister (authorization #16589 – A17034).

#### Mice

Females BALB/cByJ mice (3 weeks old, Centre d’Elevage René Janvier, Le Genest Saint-Isle, France) were housed in filtered cages under normal SPF husbandry conditions and received a standard diet (LASQCdiet^®^ Rod16-R, Genobios, Laval, France; 16.9% of proteins) deprived of animal proteins, in which no BLG was detected using specific immunoassays (29). Mice were acclimated for 2 weeks before experimentation. Three days before starting the experiments, mice were randomly allocated to cages corresponding to experimental groups (3–8 mice/cage; see below) and individually identified by ear tattooing. No difference in mean weights was observed between groups (not shown).

#### Administrations and Samplings

**FIGURE 1:**
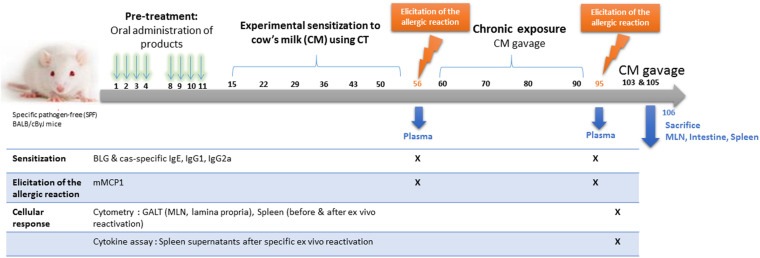
Experimental schedule.

The schedule of the experimental protocol is provided Figure 1. Mice received one intra-gastric gavage per day (200 μl/gavage) on days 1, 2, 3, 4 and 8, 9, 10, and 11 with either phosphate buffer saline (PBS, positive control of sensitization), a PBS solution containing eHC, a PBS solution containing eHC plus LGG (10^8^ CFU/100 g, similar to ratio in Nutramigen LGG formulation), or a PBS solution containing MPI. Ten mg of CMP were administered by gavage in eHC+/-LGG and MPI groups, corresponding to 1–2% of the total protein intake provided by the standard diet, which was considered as negligible. Administrations were performed following doses and protocol that favor oral tolerance induction (26), using an animal feeding needle (Popper & Sons, New Hyde Park, NY, United States).

After these pretreatments, all mice were submitted to a protocol of experimental sensitization to cow’s milk proteins (CMP, i.e., to both caseins and whey proteins), which consisted of repeated administrations of 180 μl of whole CM (eq. to 6 mg proteins/gavage) mixed with 20 μl of the Th2 mucosal adjuvant *Cholera Toxin* (10 μg/mice; Sigma Aldrich, St. Louis, United States) (20). Administrations were performed once a week, for 6 weeks (i.e., on days 15, 22, 29, 36, 43 and 50). On day 56, a first OFC was performed with 20 mg of CMP, and plasma was obtained 3 h later to assess antibodies and mouse mast cell protease-1 (mMCP-1) concentrations (see below). Additional gavages with CM (200 μl) were performed on days 60, 70, 80, and 90 to assess the persistence of any tolerogenic effects upon a chronic exposure. A second OFC was performed on day 95, and plasma collected as previously. One week after, two additional gavages with 200 μl of CM were performed (days 103 and 105). On day 106, mice were finally sacrificed and spleen, mesenteric lymph nodes (MLN) and small intestine were collected in PBS-Glucose (1 g/l) to analyze cellular responses. The group of naïve mice only received the OFCs. All collected samples (plasma, organs) were identified and treated individually.

#### Experimental Groups

Two separate protocols were conducted (T1: eHC; T2: eHC+LGG) (Table 1). For each protocol, two independent experiments (A and B) were performed in parallel, 2 to 3 weeks apart, to assess the reproducibility of any observed effects. In each protocol, 16 mice received PBS (positive control of sensitization), 10 mice received eHC (T1) or eHC+LGG (T2), and 5 mice received MPI as pretreatment. In parallel, six mice were kept naïve (neither pre-treated nor experimentally sensitized to CMP).

**TABLE 1:** Protocols and subgroups.

Protocol	Sub-groups	Gavage pre-treatment	Experimental sensitization	Number of mice
T1	A	PBS	Cow’s milk + CT	8
		eHC	Cow’s milk + CT	5
		MPI	Cow’s milk + CT	5
		Naive	PBS	3
	B	PBS	Cow’s milk + CT	8
		eHC	Cow’s milk + CT	5
		Naive	PBS	3
T2	A	PBS	Cow’s milk + CT	8
		eHC+LGG	Cow’s milk + CT	5
		MPI	Cow’s milk + CT	5
		Naive	PBS	3
	B	PBS	Cow’s milk + CT	8
		eHC+LGG	Cow’s milk + CT	5
		Naive	PBS	3

*Detailed protocol is provided Figure 1.*

### Analysis of the Humoral Response

BLG- and caseins (Cas)-specific IgE, IgG1, and IgG2a antibodies were assayed as previously described using allergen-coated microtiter plates (26, 30). For IgG1 and IgG2a, standard curves were performed on each assay plate using mixes of purified and standardized BLG- or Cas-specific monoclonal antibodies produced and characterized in the lab. Results are then provided as ng/mL. For specific IgE, serial dilution of a pool of hyper-immune plasma was used as a standard on each assay plate. Results are then provided as “Arbitrary Units.”

### Elicitation of the Allergic Reaction

Mouse mast cell protease 1 was assessed as a marker of the elicitation of an immediate intestinal allergic reaction, using commercial kit (Mouse mMCP-1 ELISA Ready-SET-Go!, Affymetrix, eBioscience, San Diego, CA, United States) following the provider’s recommendations. No clinical symptoms were evidenced in BALB/c mice when performing sensitization with cholera toxin and an OFC with 20 mg of CMP.

### Analysis of Cellular Responses

#### Extraction and Reactivation of Spleen Cells

After mechanical dilaceration of the spleen (Gentle MACs dissociator, Miltenyi Biotec GmbH, Bergisch Gladbach, Germany), red blood cells were lysed (Red Blood cell Lysis Buffer, Sigma). Splenocytes were then washed and finally suspended in RPMI-10 (RPMI supplemented with 10% fetal calf serum (FCS), 2 mM L-glutamine, 100 U penicillin, 100 μg/ml streptomycin; all from GIBCO^®^, Thermo Fisher Scientific, Waltham, United States). After numeration and assessment of viability using 7-amino-actinomycin D (7-AAD, Life technologies, Carlsbad, United States), cell concentrations were adjusted. Part of cells were used for T helper (Th) and regulatory T (Treg) cells labeling (see below). Other spleen cells were labeled with CFSE (CFSE Cell Division Tracker Kit, Biolegend, San Diego, United States) following the provider’s recommendation. Cells were then dispatched in 96-well culture plates (10^6^ cells/well), and purified BLG or Cas [(31); final concentration 20 μg/ml] were added to activate specific memory T cells. Purified proteins were pre-incubated with polymyxin (Sigma-Aldrich, final concentration 50 μg/ml) in order to neutralize any LPS contamination. Efficiency of neutralization was confirmed by the fact that neither cell proliferation nor cytokine secretion was evidenced in spleen cell from naïve mice cultured with BLG or Cas. Concanavalin A (1 μg/ml) was used as a positive control of activation, and RPMI-10 as a negative control (not shown). After incubation for 60 h at 37°C (5% CO2) and centrifugation (300 *g*, 10 min, +4°C), the supernatants were collected and stored at −80°C, and cells were collected for Treg/Th cell staining (see below). IL-5, IL-13, IL-10, IFNγ, and IL-17 cytokines were assayed by multiplexed assays on undiluted supernatants using apparatus and commercial kits from BioRad (BioPlex200^®^, BioRad, Marnes-la-Coquette, France), following the provider’s recommendations.

#### Cell Extraction From MLN and Lamina Propria

Cell suspension was obtained from MLN after manual dissociation on a cell strainer (70 μm; BD, Le Pont de Claix, France). Small intestine was collected and flushed with 10 ml of PBS. After Peyer’s patches removal, cells were extracted from lamina propria (LP) by successive incubations in HBSS, 2 mM EDTA, 10 mM HEPES, and extracellular matrix digestion (RPMI, 10 mM HEPES, 25 μg/ml Liberase (Roche, Sigma; 0.13 WU), 10 U/ml DNAse I). Numeration and viability were assessed by flow cytometry using 7-AAD, and cell concentrations adjusted in PBS, 1 mM EDTA, 2% FCS for staining.

#### Cell Staining

5 × 10^5^ cells were stained for Th or Treg using the following anti-mouse antibodies (all from BioLegend, except when specified). *Treg:* PE anti-Foxp3, PerCP/Cy5.5 anti-Helios, PE/Cy7 anti-CCR9, AlexaFluor647 anti-CD39, APC/Fire750 anti-CD45, BV421 anti-LAP, BV510 anti-CD4, BV605 anti-CTLA4, and BV785 anti-CD25. *Th:* PE anti-Foxp3, APC anti-RORγt (eBioscience), PE/Cy7 anti-CCR9, BV421 anti-GATA3, BV605 anti-Tbet, APC/Fire750 anti-CD45, BV510 anti-CD4, and BV785 anti-CD3. All antibodies were first titrated for optimal dilution (0.1–2 μg/ml for 10^6^ cells). FcR were blocked using anti-CD16/anti-CD32 (2.4G2, BD Pharmingen, Le Pont de Claix, France), and cells were incubated with antibodies for extracellular labeling for 30 min at +4°C. After washing, cells were fixed and permeabilized (True-Nuclear Transcription factor buffer set kit, Biolegend). After a new incubation with anti-CD16/CD32 antibodies, intracellular staining (Foxp3, Tbet, RORγt, GATA-3, and Helios) was performed for 45 min at +4°C. Compensations were performed using beads (UltraComp eBeads; Life technologies) stained with the same antibodies.

All acquisitions were performed on a Novocyte 13-colors flow cytometer (ACEA Bioscience, Inc., San Diego, CA, United States). Analysis was performed through FlowJo^®^ v10 (FlowJo LLC, Ashland, OR, United States). We first combined analysis of extracellular markers (CD45, CD3, CD4, and CCR9 for intestinal homing) to that of transcription factors (T-bet, GATA-3, RORγt, and Foxp3) to have an overview of Th and Treg cells induced in the intestine. For a more in-depth analysis of Treg cells, we also analyzed Foxp3, Helios, LAP, CTLA-4, CCR9, and/or CD39 expression within CD4^+^CD45^+^ gated cells. Helios^–^Foxp3^+^ cells were defined as “iTreg” (Treg induced in periphery against exogenous antigen) and Foxp3^–^LAP^+^ cells as “Th3” cells (32).

### Statistical Analysis

#### Assessment of Data Homogeneity for a Same Pretreatment Between Subgroups and Protocols

For mice receiving the same pretreatment, homogeneity of data obtained in the two protocols (PBS and MPI) and/or in the different sub-groups (i.e., eHC, eHC+LGG) was checked for each analyzed variable (i.e., all humoral and cellular data, mMCP1 concentrations) [Rcmdr package and “coin” plugin, script for reiteration of oneway_test and adjustment for multiple testing using false discovery rate (fdr), R software]. If no difference was evidenced between subgroups and/or between protocols for a given variable, all data corresponding to this variable were gathered by pretreatment. Conversely, data from protocols or sub-groups were analyzed separately if a significant difference was evidenced.

Thanks to this first analysis, we were able to gather all data obtained for a same pretreatment from the different subgroups and protocols for BLG- and Cas-specific IgE, IgG1, and IgG2a antibodies concentrations and mMCP1 concentrations. Conversely, we observed significant differences for cytokine concentrations for a same pretreatment between protocols and between subgroups. We then expressed each cytokine as a percentage, with PBS pretreated mice taken as an internal reference within each subgroup (100%). Once expressed this way, no statistically significant difference was evidenced for a same pretreatment between protocols and/or subgroups, allowing corresponding data to be gathered. All these gathered data (specific antibodies and cytokines concentrations, mMCP1 concentrations) were then aggregated to perform multivariate analysis (see below), and classical univariate analysis.

For cytometry analysis, a higher heterogeneity was observed between the experiments. We gathered data or had to analyze the data protocol per protocol, or even subgroup per subgroup, depending on the population or organ considered (see section “Results”). Data from cytometry were then analyzed independently from other data using univariate analysis (see below).

#### Multivariate Analysis

Firstly, we performed a descriptive analysis through a principal component analysis (PCA) of all the aggregated data (antibodies, cytokines and mMCP1 concentrations) obtained from each individual to have an overview of all the individuals, to identify potential outliers (none identified), and to assess the variables which are the most explicative of the whole dataset. Non-supervised clustering was also tested (Hierarchic Classification on Principal Components, HCPC; R software, FactoMineR plugin); HCPC gathers the individuals that are closer when considering all the variables, without any *a priori*: if pretreatments have no effect, individuals will then be homogeneously shared into the different clusters, which is assessed via a chi-square test.

Then, we modeled all the aggregated data (antibodies, cytokines and mMCP1 concentrations) using supervised Partial Least Square-Discriminant Analysis (PLS-DA^®^, XLSTAT software, Addinsoft, Paris, France), with pretreatment identified as the explicative variable (PBS, eHC; eHC+LGG or MPI). If such a model is successfully constructed, that means that it is possible to classify the mice depending on the pretreatment they received thanks to the analyzed components, and then that each pretreatment may have a specific effect. Such a model will then allow identifying the “discriminant variables”, that is to say the set of components that mainly participated in the model construction and then that mainly supported the differences between the groups. Those components are identified thanks to model-calculated variable important in projection values (VIP), and are selected as showing VIP ± SD > 1.

#### Univariate Analysis

For a given variable, all groups were compared to all others using pairwise comparison (permutation *t*-test with false discovery rate (fdr) adjustment; R software, RVAideMemoire package). When specified, we also compared all the groups to the PBS group only (non-parametric Kruskal–Wallis and Dunn’s post-test, GraphPad Software, San Diego, CA, United States). A *p* < 0.05 value was considered significant. A trend was noticed for *p*-value 0.05 < *p* < 0.1.

## Results

### Sensitization and Elicitation of the Allergic Reaction to CMP in Pretreated Mice

Comparable results were obtained after the sensitization (day 56; Figure 1: specific antibodies and mMCP1 concentrations) and after the chronic exposure (specific antibodies and cytokine secretion, mMCP1 concentrations). For clarity, only the later results will be presented in the following.

#### Multivariate Analysis of the Humoral and Cellular (Cytokines) Parameters

We first performed a descriptive non-supervised analysis (PCA) of the seventeen variables obtained from each individual and that we can gather after the second OFC (BLG and Cas-specific IgE, IgG1 and IgG2a antibodies concentrations, BLG and Cas-specific IL-5, IL-13, IL-10, IL-17, and IFNγ secretions, mMCP1 concentrations; Supplementary Figure S1). This analysis highlighted that BLG and Cas-induced IL-5, IL-13, and IFNγ and Cas-induced IL-10 secretions were highly correlated together and are the main contributors of first dimension of PCA, that explained 38.9% of the total variance of the whole dataset. BLG and Cas-specific IgE and IgG1 antibodies, and mMCP1 are the main contributors of the second PCA dimension (16% of total variance). Conversely, BLG- and Cas-specific IgG2a, and BLG-specific IL-10 supported few information, as shown by their low-length vectors in the PCA. Non-supervised HCPC already evidenced a pretreatment effect (*p* = 0.0035), with classification of eHC+LGG mice in a separate cluster (not shown).

Data modeling using supervised analysis (PLS-DA) of the 17 variables led to the construction of a 2-components model with low predictive values (R^2^X cum = 0.516, R^2^Y cum = 0.171). Actually, only PBS and eHC+LGG pre-treated mice were correctly classified, in two separate groups. This suggests that these mice are not comparable for the global information provided by the 17 variables analyzed. Conversely, eHC mice were classified in the same group as PBS mice, suggesting that PBS and eHC mice are comparable for the global information provided by the 17 variables. BLG and Cas-specific IL-5 and IL-13, anti-BLG IgG1, mMCP-1, and Cas-specific IL-10 were identified as the discriminant variables of the PLS-DA (VIP ± SD > 1; Supplementary Table S1), i.e., as the variables that mainly supported the differences identified between the groups.

#### Univariate Analysis of the Humoral and Cellular (Cytokines) Parameters

**FIGURE 2:**
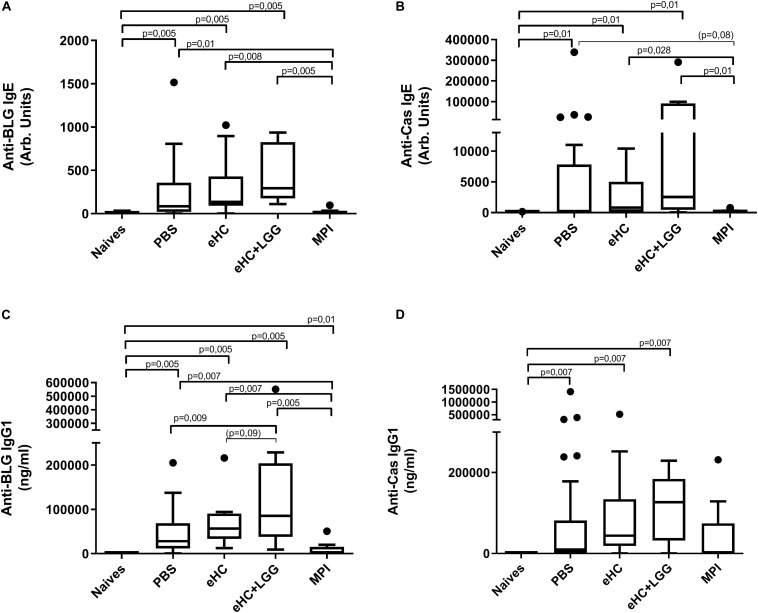
Anti-BLG and anti-Cas IgE [**(A,B)**, respectively] and IgG1 [**(C,D)**, respectively] antibodies concentrations in mice receiving gavage with PBS (*n* = 32), eHC (*n* = 10), eHC+LGG (*n* = 10), or MPI (*n* = 10) before the oral sensitization to cow’s milk proteins. Naïve mice (*n* = 12) were not treated nor sensitized, but were challenged. Blood samples were obtained after the chronic exposure to CM and 3 h after a second OFC (day 95). Medians (bars) with box and Tukey whiskers are shown for each treatment group. All groups were compared to each other using pairwise comparison and permutation *t*-test; corresponding fdr-adjusted *p*-values are indicated. Trend (0.05 < *p* < 0.1) and associated *p*-value are indicated into brackets.

**FIGURE 3:**
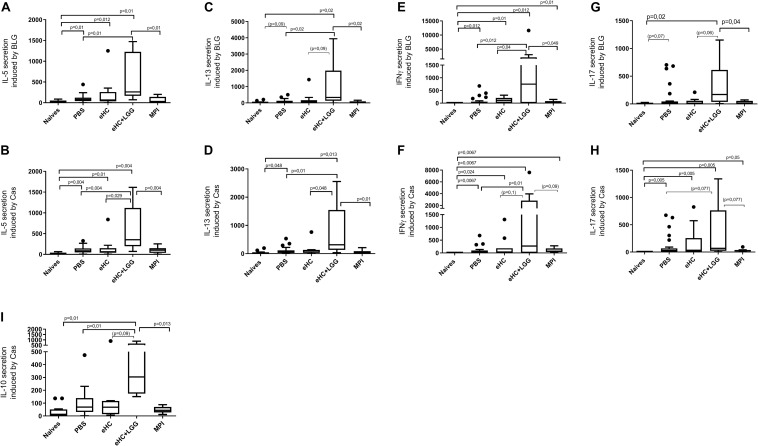
BLG and Cas-induced secretion of IL-5 [**(A,B)**, respectively], IL-13 [**(C,D)**, respectively] IFNγ [**(E,F)**, respectively], and IL-17 [**(G,H)**, respectively], and Cas-induced secretion of IL-10 **(I)** in mice that received gavage with PBS (*n* = 32), eHC (*n* = 10), eHC+LGG (*n* = 10), or MPI (*n* = 10) before the oral sensitization and then chronic exposure to CM. Naïve mice (*n* = 12) were not treated nor sensitized. Cytokines were assayed in supernatants obtained from individual spleen cells stimulated *ex vivo* with purified BLG or Caseins. Results are expressed as percentage of secreted cytokines using PBS group as an internal reference within each sub-groups (100%). Medians (bars) with box and Tukey whiskers are shown for each treatment group. All groups were compared using pairwise comparison and permutation *t*-test; corresponding adjusted *p*-values are indicated. Trend (0.05 < *p* < 0.1) and associated *p*-value are indicated into brackets.

**FIGURE 4:**
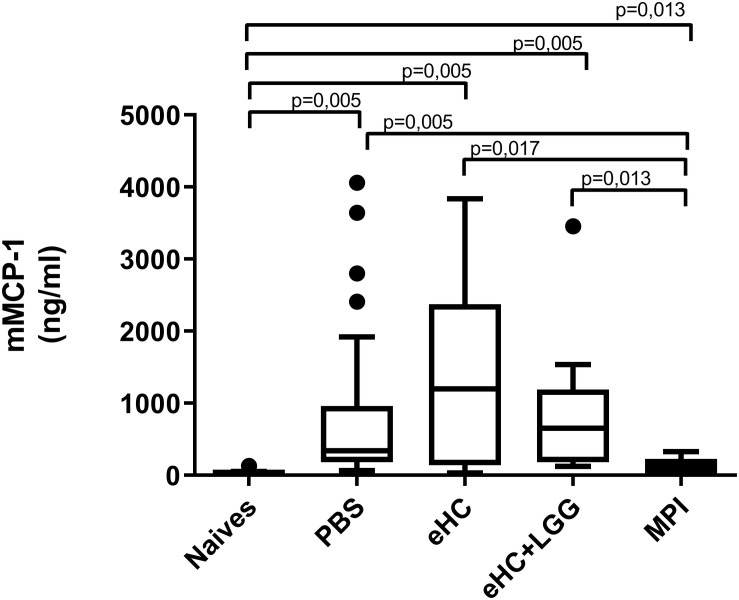
mMCP1 concentrations in plasma from mice pretreated with PBS (*n* = 32), eHC (*n* = 10), eHC+LGG (*n* = 10), or MPI (*n* = 10) before the oral sensitization, then chronically exposed to CM. mMCP1 was assessed 3 h after an OFC with 20 mg of CMP. Naïve mice (*n* = 12) were not treated nor sensitized, but were challenged. Medians (bars) with box and Tukey whiskers are shown for each treatment group. All groups were compared to each other using pairwise comparison and permutation *t*-test; corresponding fdr-adjusted *p*-values are indicated.

In parallel, we performed univariate analysis and graphically represented the data to visualize differences between groups. Anti-BLG and anti-Cas IgE and IgG1 antibodies were significantly induced in PBS-pretreated and CMP-sensitized mice compared to naïve mice (Figures 2A–D), which was associated with significant secretion of Th2 cytokines (IL-5 and IL-13) upon BLG and Cas *ex vivo* stimulation, and with significant secretion of Th1 (IFNγ), Th17 (IL-17) and regulatory (IL-10) cytokines, mainly upon Cas re-stimulation (Figures 3A–I). In line with this high sensitization status of PBS-pretreated mice, OFC induced a significant increase of mMCP1 concentrations in plasma (Figure 4), traducing the elicitation of an allergic reaction in these mice. Conversely, gavage with non-hydrolyzed CMP (MPI pretreatment group) significantly prevented CMP allergy, as evidenced by decrease of specific IgE and IgG1 concentrations (Figures 2A–D) and prevention of the elicitation of the allergic reaction (Figure 4) compared to PBS-pretreated mice. This was associated with absence of Th2 and IL-10 cytokines secretion, although low but significant secretions of IFNγ and IL-17 were still observed (Figure 3).

In line with multivariate analysis, PBS and eHC-pretreated mice were comparable for all the analyzed parameters, i.e., BLG and Cas-specific antibodies (Figure 2) and cytokines (Figure 3), and mMCP1 release after OFC (Figure 4). CMP allergy was also significantly induced in eHC+LGG pretreated mice. However, eHC+LGG pretreated mice had significantly higher BLG-specific IgG1 antibodies concentrations compared to all other groups (Figure 2C). A significant/trend increase of anti-BLG (*p* = 0.03) and anti-Cas (*p* = 0.1) IgE antibodies concentrations was also observed in eHC+LGG pretreated mice when comparing all groups to the PBS one. BLG and Cas-induced IL-5, IL-13, IFNγ, and IL-10 secretions were also significantly increased in eHC+LGG compared to PBS and (for some) to eHC pretreated mice (Figure 3).

### Analysis of Th and Treg Cells in Gut Associated Lymphoid Tissue (GALT) and in Spleen

No significant difference was observed in Th and Treg cell subpopulations frequencies analyzed in the MLN or spleen at sacrifice (not shown).

#### Lamina Propria

**FIGURE 5:**
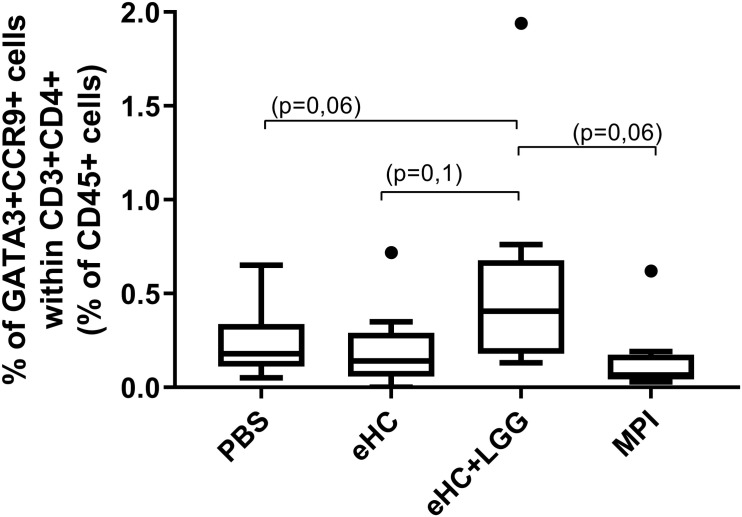
Percentage of Th2 CCR9+ cells in LP from mice of the different treatment groups. Aggregated data from the two protocols are shown. A first gate was designed based on structural parameters (FSC and SSC), and then single cells were selected (FSC-A × FSC-H). Within single cells, CD45^+^FSC^low^ cells were gated, in which we selected CD3^+^CD4^+^ T cells. Within these cells, intestinal Th2 cells were identified thanks to the co-expression of transcription factor GATA3 and homing receptor CCR9. Medians (bars) with box and Tukey whiskers are shown for each treatment group. All groups were compared to each other using pairwise comparison and permutation *t*-test; corresponding adjusted *p*-values are indicated.

A trend in increased frequency of RORγt^+^Foxp3^+^ cells was noticed in LP from eHC pretreated mice (*p* = 0.09 versus PBS, MPI and eHC+LGG mice; FDR-adjusted value from pairwise permutation test; not shown). Conversely, a reproducible significant decrease of CCR9^+^CD39^+^ cells within CD4^+^Foxp3^+^ Treg cells in LP from eHC compared to PBS pretreated mice was observed (intra-protocol analysis, not shown). In parallel, a trend in increased frequency of CCR9+Th2 cells was observed in LP from eHC+LGG pretreated mice compared to other pretreated groups (Figure 5), in line with the higher sensitization status of these mice.

#### Spleen Cells After *ex vivo* Reactivation

**FIGURE 6:**
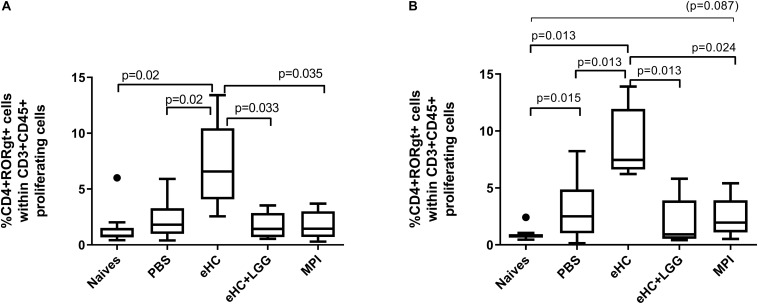
Percentage of CD4+RORγt+ cells within proliferating cells after specific *ex vivo* reactivation with BLG **(A)** or Caseins **(B)**. A first gate was designed based on structural parameters, and then single cells were selected. Within single cells, CFSE^low^ cells (i.e., proliferating cells) were gated and then analyzed for CD3 and CD45 expression. Within CD3^+^CD45^+^ proliferating cells, co-expression of CD4 and transcription factors RORγt was assessed. Comparable results were obtained when selecting first CD45^+^CFSE^*low*^ within single cells, then gating CD3^+^CD4^+^ cells and analyzing expression of transcription factors within this latter population. Medians (bars) with box and Tukey whiskers are shown for each treatment group. All groups were compared to each other using pairwise comparison and permutation *t*-test; corresponding adjusted *p*-values are indicated. Trend (0.05 < *p* < 0.1) and associated *p*-value are indicated into brackets.

Analysis of splenocytes after specific *ex vivo* stimulation showed a comparable percentage of proliferating cells (CFSE^low^) within CD45^+^CD4^+^ cells in CMP sensitized mice (not shown). The percentage of CD4^+^RORγt^+^ Th17 cells significantly increased in the eHC group after BLG and/or caseins *ex vivo* stimulation (Figures 6A,B). We also observed an increased frequency of CD4^+^GATA3^+^ Th2 cells in eHC mice compared to PBS mice after BLG *ex vivo* stimulation, which was associated with a decrease of CD4^+^Foxp3^+^ frequency (intra-protocol analysis; not shown). No significant change was noticed in eHC+LGG group.

## Discussion

The aim of the present study was to assess the effect of administration of an extensive hydrolysate from caseins (eHC), supplemented or not with LGG probiotic, on the further experimental induction of CMP allergy. Thanks to a validated mouse model of CMP allergies, both specific and non-specific effects of casein-derived peptides were assessed.

We evidenced that, as expected, a CMP allergy is efficiently induced in PBS-pretreated and CMP-sensitized mice, as shown by high specific IgE and IgG1 antibody concentrations, high specific Th2 cytokine secretion and high mMCP1 concentrations after OFCs. Conversely, gavage with non-hydrolyzed CMP (here MPI) efficiently prevent further induction of CMP allergy. No protective effect of eHC+/-LGG on the sensitization to casein could be evidenced, nor on sensitization to other non-related CMP (here the whey protein BLG), and no protection was provided on elicitation of the allergic reaction to CMP. Although eHC mice could not be distinguished from PBS groups for all analyzed parameters, eHC+LGG mice were characterized by enhanced humoral and cellular immune responses, both to caseins and BLG.

Firstly, we would like to point out that we uniquely analyzed our data through rigorous statistical procedures: (i) assessment of homogeneity of data between protocols and subgroups allowing (or not) to gather data and then to increase statistical power, (ii) descriptive analysis (PCA and HCPC) of gathered data further aggregated, in order to identify potential outliers within the individuals and the most contributive variables in the global response, but also to anticipate differences between groups, and (iii) supervised analysis to identify differences (or their absence) between groups and the variables supporting these differences. Univariate analysis (with correction for multiple testing) allowed comforting these results and visualizing the differences between groups. Such statistical procedure in experimental models may improve the quality, rationalization and robustness of *in vivo* studies that integrate several parameters on the same animal and that aim to compare different (pre)treatments.

Concerning the results obtained with eHC alone, our results are in line with, and extend previous results demonstrating the high and specific prevention potency of non-hydrolyzed CMP (here MPI), and the loss of efficiency of this preventive specific effect while the degree of hydrolysis increases (18, 23, 25, 26). In line with these results, a mix of four 18 amino-acid long synthetic peptides derived from BLG administered orally before oral sensitization to CMP did not prevent a local or systemic CMP allergy (33). In another model, eHW given for 3 weeks through the drinking water (∼180 mg of proteins/day) had no effect on epicutaneous sensitization to BLG, but an attenuation of anaphylaxis and activation of intestinal mast cells was observed after an OFC (34). We then cannot exclude that a longer pretreatment period and higher doses of eHC would have a significant effect on sensitization or elicitation to caseins in our experimental setup. However, 180 mg of whey protein for a 20 g mouse is equivalent to 54 g of protein for an infant of 6 kg. As infant formulas contain 1.3–1.4 g of protein per 100 ml, the quantity of formula ingested by the baby would be 3.8–4.1 L/day.

Alternatively, Aitoro et al. (27) reported prevention from allergy to purified BLG by eHC administration, an effect that then results from non-specific bioactivity of peptides derived from caseins. Discrepancies between this later study and ours should not rely on eHC composition that demonstrated minor batch-to-batch variations (28). In Aitoro’s study, eHC was administered through the drinking water as the sole source of food, and was compared to a standard solid diet. However, intervention and standard diets were not comparable for the protein load but also for nutrients such as dietary fibers, fatty acids, vitamin D and folic acids. Those components can critically affect the intestinal barrier, the immune system and the composition and function of the intestinal microbiota, all of which influence a further experimental allergic sensitization. Moreover, they pursued eHC administration during sensitization with BLG and cholera toxin (CT), whereas κ-casein derived glycomacropeptides have been described to inhibit binding of CT to its receptor, at least *in vitro* (35, 36). Glycomacropeptides is hydrolyzed in eHC, but some derived peptides (37) may still interfere with CT and then with the experimental sensitization to BLG. Such non-specific effects could not be evidenced in our experimental setup since eHC administration was not pursued during sensitization.

Considering the cellular responses, we observed a trend in increased frequency of RORγt^+^Foxp3^+^ cells in LP, and a significant increase of RORγt^+^ and GATA3^+^ cells among proliferating splenic cells from eHC pretreated mice. Although these changes did not affect sensitization and elicitation parameters, further analysis in GALT focusing on these parameters just after the pre-treatment phase would be instructive. RORγt^+^Foxp3^+^ cells are regulatory cells of importance in the intestine that participate in inflammation control and are induced for example by probiotic strain (38).

Our present study also revealed the strong immunostimulatory potential of LGG. We observed a significant increase of almost all immune parameters in eHC+LGG pretreated mice compared to PBS or eHC pretreated mice. It is worth noting that cellular response differences were mainly revealed through cytokine secretion: small differences were observed through deep cytometry analysis on GALT and spleen cells, even after *ex vivo* restimulation. This thus suggests that the activity (i.e., secretion capacity) rather than the increased frequency or proliferation of specific subpopulations is detectable in our experimental setting. Moreover, despite an increase of specific-antibodies concentrations in eHC+LGG pretreated mice, we did not evidence an increase of mMCP1 concentrations after the OFCs, which would require further investigations (e.g., comparison of mast cell density and FcγRI expression in intestine). Our results are then in contradiction with most of the studies available. For example, more significant preventive (and therapeutic) effects were reported when using eHC+LGG compared to eHC in the BLG-allergy model (27), in line with the clinical results obtained in CMP allergic patients (8). It is clear that the administration of LGG before sensitization (preventive strategy) will not have the same effect than administration of the same compounds in an already sensitized organism (therapeutic strategy). In the therapeutic schedule, Th1/Th17 induced response (as evidenced in our experiments by increased IFNγ and IL-17 secretion in eHC+LGG group) may rather counteract the on-going Th2 immune response, as observed in clinical trials (3, 8, 9). IL-10 induced in the eHC+LGG group may also play a more pronounced regulatory role in this context. But, in the preventive strategy, the time lapse between LGG and sensitizing administration may also be of importance. Actually, transient modification of the gut microbiota composition (unfortunately not assessed in our experiments) and the immune response potentially induced by LGG may amplify the adjuvant effect of CT, or on the contrary repress it, depending on the immune status at the exact moment CT is administered (i.e., “inflammation burst” versus “inflammation resolution”). Further studies combining non-hydrolyzed proteins [e.g., MPI or purified BLG (23, 26)] plus LGG intervention should be conducted to further assess the immunostimulatory effect of LGG and its effect on the induction of oral tolerance. Other probiotics strains should be tested as well in a comparable experimental model.

## Conclusion

In conclusion, we could not evidence any preventive effect, either specific or non-specific, of administration of extensive hydrolysates from caseins on further experimental CMP allergy. The pre-clinical data we provide are in line with others, and a with recent population-based study that did not observe preventive effect of the use of pHF at 2 months on food allergy, both in at risk and non-at risk infants (15). Altogether, these results then further challenge the use of hydrolysates for allergy prevention. Unexpectedly, we also evidenced that co-administration of LGG with eHC enhanced the immune response induced against CMP. Our results do not challenge the efficiency of eHC supplemented with LGG as a therapeutic strategy for allergic infants evidenced in clinical trials (3, 8). However, and although our findings obtained in a mouse model cannot be translated directly to weaning neonate/infants, further studies in a preventive set up should be conducted to further analyze the effect of early nutritional intervention using LGG on food allergy development, independently of hydrolysates, to understand immune mechanisms involved, and to clarify their significance in clinical applications.

## Data Availability Statement

The datasets generated for this study are available on request to the corresponding author.

## Ethics Statement

The animal study was reviewed and approved by French Minister (authorization #16589 – A17034).

## Author Contributions

KA-P designed the whole study, analyzed and interpreted the data, and wrote the manuscript. KA-P, MG, BG, HB, and AC performed the experiments. SH performed some experiments and critically revised the manuscript. CJ critically revised the manuscript. All authors approved the submitted version.

## Conflict of Interest

The authors declare that the research was conducted in the absence of any commercial or financial relationships that could be construed as a potential conflict of interest.

## Abbreviations

BLG: bovine β -lactoglobulin
Cas: caseins
CMP: cow’s milk proteins
CT: Cholera toxin
eHC: extensive hydrolysates from caseins
eHW: extensive hydrolysates from whey
HCPC: Principal Component Analysis and Hierarchic Classification on Principal Components
LGG: *Lactobacillus rhamnosus* GG
LP: lamina propria
MLN: mesenteric lymph nodes
mMCP1: mouse mast cell protease 1
MPI: non-hydrolyzed milk proteins isolate
OFC: oral food challenge
PCA: principal component analysis
pHW: partial hydrolysates from whey
PLS-DA: partial least square – discriminant analysis
VIP: variable important in projection.
