# Supplementary material for: Administration of Extensive Hydrolysates From Caseins and Lactobacillus rhamnosus GG Probiotic Does Not Prevent Cow’s Milk Proteins Allergy in a Mouse Model
Source: Front Immunol. 2020 Sep 11;11:1700. doi: 10.3389/fimmu.2020.01700 (PMC7516991; doi:10.3389/fimmu.2020.01700)
Supplement: Supplementary file 1 [file Table_1.DOCX]

Administration of extensive hydrolysates from caseins and *Lactobacillus rhamnosus GG* probiotic does not prevent cow’s milk proteins allergy in a mouse model

Karine Adel-Patient, Marine Guinot, Blanche Guillon, Hervé Bernard, Amina Chikki, Stéphane Hazebrouck and Christophe Junot

**Supplementary files**

**
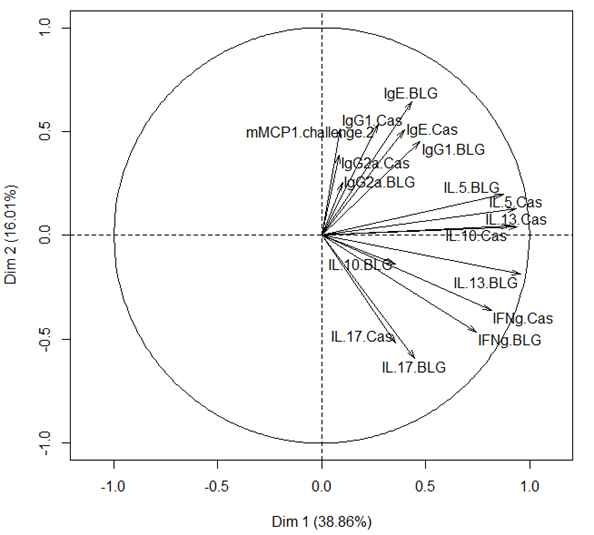
Suppl figure 1:** Non-supervised multivariate analysis (PCA) of the seventeen variables measured for each individuals after the second OFC. Graph of variables is shown, with percentage of total variance explained on dimension 1 (x-axis) and dimension 2 (y axis) indicated.

**Supplementary table 1:** VIP values obtained for the 17 aggregated variables. A 2-dimension PLS-DA was constructed with the pre-treatment group as explicative variable. Bold indicated VIP values +/- SD > 1.

| **Variable** | VIP-1 | SD | VIP-2 | Ecart-type |
| --- | --- | --- | --- | --- |
| IL-5-Cas | **1,508** | 0,109 | **1,304** | 0,096 |
| mMCP1 | 0,435 | 0,301 | **1,273** | 0,305 |
| IL-13-Cas | **1,408** | 0,140 | **1,267** | 0,102 |
| IgG1-BLG | **1,298** | 0,262 | **1,240** | 0,291 |
| IL-13-BLG | **1,315** | 0,125 | **1,199** | 0,105 |
| IL-10-Cas | **1,396** | 0,196 | **1,183** | 0,180 |
| IL-5-BLG | **1,380** | 0,149 | **1,176** | 0,146 |
| IgE-BLG | 0,826 | 0,259 | 1,091 | 0,184 |
| IFNg-Cas | 1,134 | 0,155 | 1,059 | 0,147 |
| IFNg-BLG | 0,980 | 0,271 | 0,950 | 0,190 |
| IL-17-Cas | 0,884 | 0,419 | 0,867 | 0,181 |
| IL-17-BLG | 0,901 | 0,401 | 0,776 | 0,253 |
| IgG1-Cas | 0,265 | 0,409 | 0,703 | 0,359 |
| IL-10-BLG | 0,226 | 0,628 | 0,616 | 0,272 |
| IgG2a-BLG | 0,461 | 0,292 | 0,602 | 0,387 |
| IgE-Cas | 0,640 | 0,518 | 0,542 | 0,411 |
| IgG2a-Cas | 0,150 | 0,322 | 0,465 | 0,277 |
